# Microbial Synthesis of Precious Metal Nanoparticles and Their Applications: A Review

**DOI:** 10.3390/microorganisms14081726

**Published:** 2026-08-06

**Authors:** Shiyi Huang, Shuchang Liu, Jing Liu, Fengxin Pan, Zhenkun Shi, Shuang Zhou, Jianping Xie, Chaoyu Tian, Guozhen Wang, Ling Tan

**Affiliations:** 1 School of Life and Health Sciences, Hunan University of Science and Technology, Xiangtan 411201, China; 24010901003@mail.hnust.edu.cn (S.H.); 25010901115@mail.hnust.edu.cn (S.L.); 2309030320@mail.hnust.edu.cn (J.L.); 2409030205@mail.hnust.edu.cn (F.P.); zhoushuang@hnust.edu.cn (S.Z.); 2 Biodesign Center, Key Laboratory of Engineering Biology for Low-Carbon Manufacturing, Tianjin Institute of Industrial Biotechnology, Chinese Academy of Sciences, Tianjin 300308, China; zhenkun.shi@tib.cas.cn; 3 School of Minerals Processing and Bioengineering, Central South University, Changsha 410083, China; xiejianping@csu.edu.cn; 4 School of Chemical Engineering, Xiangtan University, Xiangtan 411105, China; tianchaoyu@xtu.edu.cn; 5 State Key Laboratory of Precious Metal Functional Materials, Kunming Institute of Precious Metals, No. 988 Keji Road, High-Tech Development Zone, Kunming 650106, China

**Keywords:** microbial synthesis, precious metal nanoparticles, biosynthesis mechanisms, nanoparticle applications

## Abstract

Precious metal nanoparticles (PMNPs), particularly silver, gold, palladium, and platinum nanoparticles, have attracted considerable attention owing to their unique physicochemical properties and broad applications in catalysis, environmental remediation, and biomedicine. Conventional physical and chemical synthesis methods often require substantial energy input, harsh reaction conditions, and generate large volumes of metal-containing wastewater, raising concerns regarding sustainability and environmental impact. Microbial synthesis provides a sustainable alternative by using microorganisms as natural biofactories to convert toxic precious metal ions into valuable nanoparticles under mild conditions. This review summarizes recent advances in the microbial synthesis of PMNPs (Bio-PMNPs), focusing on biosynthetic mechanisms in bacteria, algae, and fungi. Bio-PMNPs formation involves both extracellular and intracellular reduction processes, coupled with electron transfer mediated by reductases and other redox-active biomolecules. Functional groups present on microbial cell walls, as well as proteins, polysaccharides, enzymes, and other metabolites, play important roles in the adsorption, reduction, stabilization, and growth of nanoparticles. We further highlight the applications of Bio-PMNPs in antimicrobial activity, cancer therapy, pollutant degradation, heavy-metal removal, and catalytic enhancement of organic synthesis. Despite substantial progress, challenges remain in controlling nanoparticle size and morphology, elucidating biosynthetic mechanisms, and achieving large-scale production. Future integration of synthetic biology, metabolic engineering, and process optimization is expected to improve the controllability, stability, scalability, and biosafety of Bio-PMNPs production.

## 1. Introduction

Precious metals, including gold (Au), silver (Ag), platinum (Pt), palladium (Pd), rhodium (Rh), iridium (Ir), ruthenium (Ru), and osmium (Os), exhibit unique physicochemical properties and are widely applied in catalysis, pharmaceuticals, microelectronics, and electroplating [1]. Precious metal nanoparticles (PMNPs) with diameters below 100 nm [2] play an indispensable role in chemical catalysis [3], biomedicine [4], environmental remediation [5], chemical sensing, and electronic devices [6]. Physical synthesis methods for PMNPs, such as laser ablation, arc discharge, and physical vapor condensation [7], require highly specialized equipment and substantial energy input [8]. Chemical synthesis approaches, including chemical reduction, electrochemical synthesis, and wet chemical synthesis, often rely on toxic chemicals and may generate large quantities of wastewater containing metal contaminants [8,9].

Microbial synthesis of PMNPs (Bio-PMNPs) using microorganisms as natural nanofactories is a green and sustainable approach [10]. When precious metal ions enter microbial cells, they induce severe toxic stress, including disruption of protein conformation, inhibition of key enzymatic activities, excessive generation of reactive oxygen species (ROS), and damage to DNA and membrane integrity [11]. To survive in metal-rich environments, microorganisms have evolved sophisticated detoxification mechanisms through biosorption, bioreduction, biomineralization, and bioaccumulation. Biosorption involves the interaction between negatively charged functional groups of biomolecules and positively charged precious metal ions through electrostatic attraction and complexation [12]. This process lowers the concentration of free precious metal ions and partially prevents their further intracellular uptake. Bioreduction the coordinated action of intracellular and extracellular reductases, together with electron donors such as NADH and NADPH, to convert highly toxic precious metal ions into non-toxic nanoparticles [13]. Biomineralization involves the reaction of precious metal ions and metabolically generated anions (S^2−^, CO_3_^2−^, and PO_4_^3−^), resulting in the formation of low-solubility precipitates such as metal sulfides, carbonates, and phosphates [14]. Bioaccumulation involves the uptake of precious metal ions via transport systems, followed by binding to metallothioneins and other sulfur-containing proteins, causing the precious metals to accumulate within the cells in a non-toxic form [15]. Efflux systems further contribute to metal resistance by actively exporting excess precious metal ions through membrane transporters and ATP-driven pumps [16]. Microbial cells and their extracellular secretions contain abundant reductive components and biomolecules that not only efficiently reduce precious metal ions but also act as natural stabilizers to prevent nanoparticle aggregation [17]. In addition, intracellular reductases, cell wall structures, and other biomolecules provide nucleation sites and growth templates, thereby regulating nanoparticle size and morphology [7].

**Figure 1:**
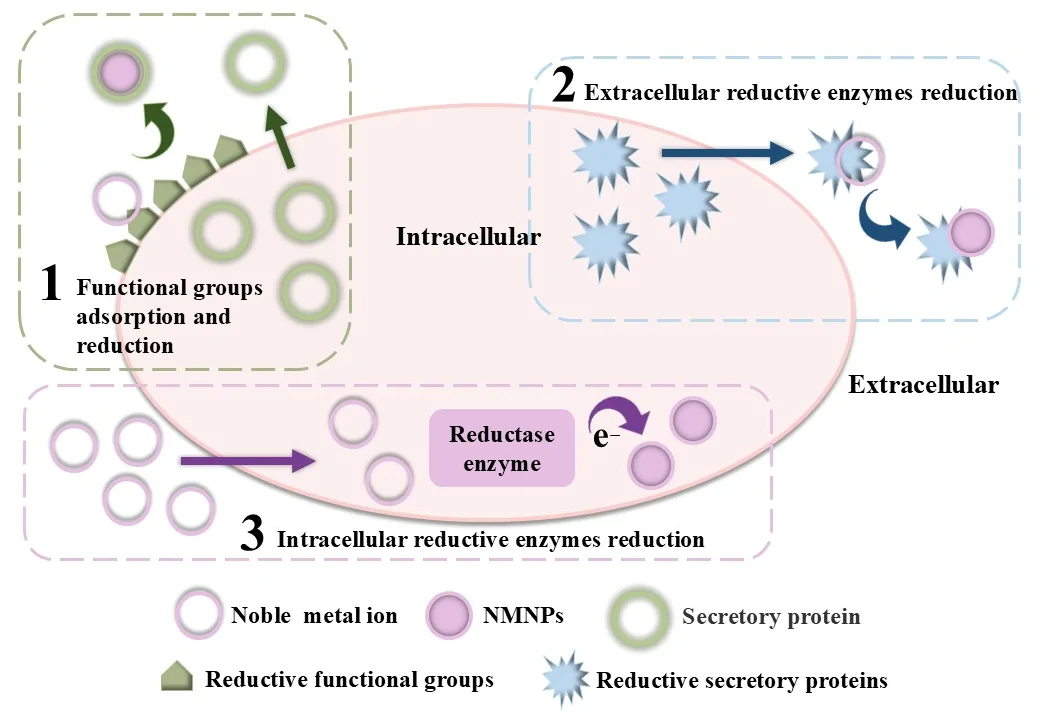
Mechanism diagram of microbial synthesis of PMNPs.

Current studies suggest that microbial synthesis of PMNPs mainly occurs through three pathways: reduction mediated by functional groups on the cell surface, reduction mediated by extracellular reductive enzymes, and reduction mediated by intracellular reductive enzymes (Figure 1). In the first pathway, precious metal ions are initially adsorbed onto functional groups on the cell wall and subsequently reduced to PMNPs by reductive moieties such as hydroxyl and aldehyde groups. Proteins, polysaccharides, and other organic compounds secreted by microorganisms further stabilize the nanoparticles [18]. In the extracellular enzyme-mediated pathway, extracellular reductive enzymes capture precious metal ions and reduce the ions at enzyme active sites, leading to the formation of PMNPs [19]. In the intracellular route, precious metal ions enter the periplasm or cytoplasm through diffusion or active transport, where reduction is mediated by intracellular reductive enzymes. The newly formed PMNPs are subsequently capped by intracellular biomolecules. Some nanoparticles are exported through efflux systems, whereas others remain inside cells as intracellular deposits [20]. Recovery of intracellularly synthesized PMNPs usually requires additional procedures, including cell disruption or biomass carbonization [21]. By contrast, extracellular synthesis facilitates separation and purification, making extracellular synthesis more favorable for large-scale production.

Compared with other metallic nanoparticles, PMNPs possess superior biocompatibility, chemical stability, and readily functionalized surfaces, rendering them particularly attractive for targeted drug delivery and cancer therapeutic applications [22]. In the field of chemical and biological sensing, PMNPs exhibit distinctive optical and electronic properties, including a high surface area-to-volume ratio, high refractive indices, and surface plasmon resonance effects. These properties collectively enhance signal transduction efficiency and improve detection sensitivity [23]. Moreover, PMNPs are widely employed as highly efficient catalysts owing to their exceptional catalytic activity and selectivity, with extensive applications in environmental remediation, pollutant degradation, and energy conversion processes [24,25].

This review summarizes the Bio-PMNPs, including Bio-AgNPs, Bio-AuNPs, Bio-PdNPs, and Bio-PtNPs mediated by bacteria, algae, and fungi. The underlying biosynthetic mechanisms are discussed, with particular emphasis on functional group-mediated reduction, enzymatic pathways, and extracellular electron transfer (EET). In addition, the applications of bio-PMNPs in catalysis, environmental remediation, and biomedicine are comprehensively reviewed.

## 2. Bio-PMNPs Synthesis

### 2.1. Bio-AgNPs Synthesis

Microorganisms, including bacteria, algae, and fungi, serve as promising biofactories for AgNPs biosynthesis through enzymatic and nonenzymatic reduction of Ag(I). During this process, microbial functional groups, biomolecules, and reductases contribute to Ag(I) reduction, while proteins and polysaccharides stabilize the generated nanoparticles by preventing aggregation. The following sections discuss the mechanisms responsible for AgNPs biosynthesis by bacteria, algae, and fungi. The major mechanisms and key reactive components are summarized in Table 1.

#### 2.1.1. Bio-AgNPs Synthesized by Bacteria

The biosynthesis of AgNPs by bacteria is mainly attributed to extracellular functional groups, biomolecules, and reductases. For example, amino and carbonyl groups in the cell-free extracts from *Bacillus pumilus*, *Bacillus paralicheniformis*, *Sphingomonas paucimobilis,* and *Bacillus lentus* contribute to the synthesis and stabilization of AgNPs [26,27]. Proteins, enzymes, peptides, polysaccharides, and organic acids in bacterial extracts, such as those from *Bacillus megaterium* [28], *Bacillus subtilis* [28,29], *Bacillus cereus* [30], and *Paenarthrobacter nicotinovorans* [31], play important roles in Ag(I) reduction and stabilization of AgNPs. However, the identities of the active reducing components in these extracts remain largely unknown, and further studies are needed to determine which molecules are directly responsible for Ag(I) reduction. Compared with functional group-based mechanisms, enzyme-mediated reduction has been investigated in greater mechanistic detail. It has been reported that nitrate reductase purified from *Bacillus clausii* can react with AgNO_3_ to generate AgNPs [32]. Structural analysis of nitrate reductase reveals that the NarG superfamily domain is responsible for ion metabolism, while molecular docking studies indicate that Ag(I) can bind to the active region of the enzyme [32]. Under normal conditions, nitrate reductase uses NADH as an electron donor to reduce NO_3_^−^ to NO_2_^−^ [50]. However, in the presence of Ag(I), the metal ion competes with NO_3_^−^ for the active site, thereby redirecting electron flow. Consequently, electrons derived from NADH are transferred to Ag(I), leading to the formation of metallic Ag(0) [51,52]. A similar enzyme-mediated mechanism for AgNPs biosynthesis was observed by Razack et al. [33] during the formation of AgNPs by *Bacillus subtilis*.

**Figure 2:**
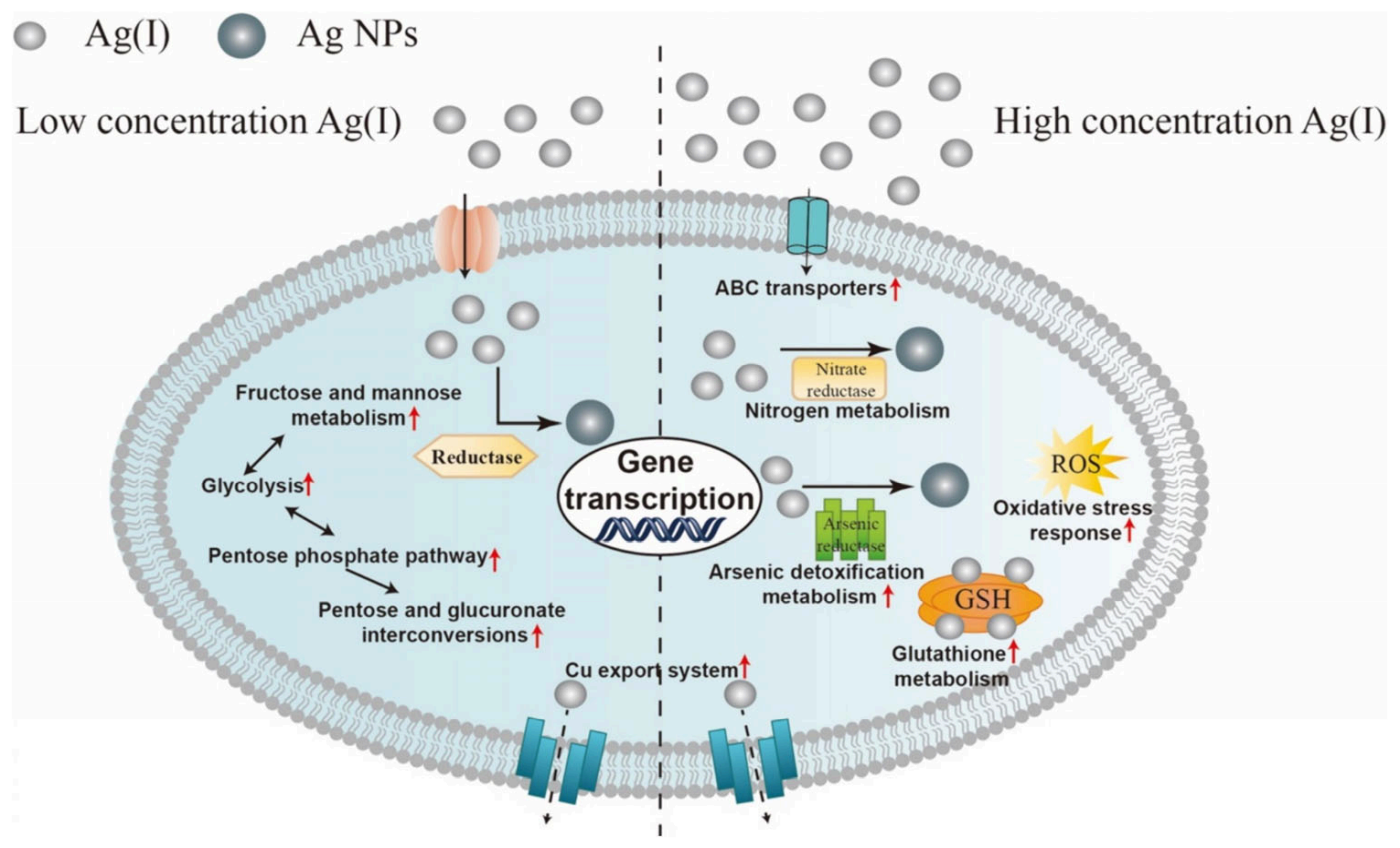
Proposed mechanistic model for microbial-mediated synthesis of AgNPs in *Pantoea* sp. under different Ag(I) concentrations. The red arrow in the figure indicates enhanced metabolism. Reprinted with permission from Elsevier. Reproduced from Liu et al. (2024) [34].

The toxicity of Ag(I) to bacterial cells results in concentration-dependent changes in cellular metabolism and detoxification responses during AgNPs biosynthesis. Liu et al. [34] integrated transcriptomic and proteomic analyses to elucidate the molecular response of *Pantoea* sp. They revealed a concentration-dependent, multi-layered strategy for Ag(I) adsorption, transport, detoxification, and bioreduction (Figure 2). Initially, Ag(I) is adsorbed onto the cell surface, where polysaccharides in extracellular polysaccharides (EPS) reduce Ag(I) through hemiacetal groups, while most Ag(I) is subsequently transported into the intracellular space. At low Ag(I) concentration (50–100 µM), cells exhibit enhanced central carbon metabolism, including fructose and mannose metabolism, glycolysis, and the pentose phosphate pathway. Consequently, the production of ATP, NADH, and NADPH is substantially increased. ATP mainly supports metal transport and cellular metabolic activity, whereas NADH and NADPH act as electron donors that drive enzymatic reduction of Ag(I) to Ag(0). At high Ag(I) concentrations (200–500 µM), intracellular accumulation of Ag(I) causes severe toxicity and activates multiple cellular defense mechanisms. Owing to the chemical similarity between Ag(I) and Cu(I), cells often misrecognize Ag(I) as Cu(I). Consequently, copper homeostasis systems are activated, including the P-type ATPase (CopA), multicopper oxidase (CueO), and Cu export systems, which reduce intracellular silver accumulation through metal binding and active efflux. Meanwhile, nitrogen metabolism is enhanced, with nitrate reductase (NarGHJI) and nitric oxide reductase (NorV) participating in electron transfer processes and utilizing NAD(P)H as electron donors. In addition, arsenic resistance gene clusters are upregulated, indicating arsenate reductase systems may contribute to Ag(I) reduction. Furthermore, the thiol group of glutathione (GSH) in cysteine residues strongly binds Ag(I), reducing free Ag(I) concentration. High Ag(I) exposure also induces severe oxidative stress and leads to excessive reactive oxygen species (ROS) production. To maintain intracellular redox homeostasis, antioxidant defense systems, including superoxide dismutase (SOD) and glutathione peroxidase, act synergistically to eliminate superoxide radicals and H_2_O_2_. The residual Ag(I) is exported from the cytoplasm, thereby minimizing toxicity and sustaining continuous AgNPs biosynthesis.

#### 2.1.2. Bio-AgNPs Synthesized by Algae

The biosynthesis of AgNPs by algae is primarily attributed to functional groups present in polysaccharides and proteins, which act as both reducing and stabilizing agents. Carboxyl, hydroxyl, carbonyl, and amide groups are involved in Ag(I) reduction and stabilization of AgNPs [35,36]. For example, sulfated polysaccharides from *Ulva armoricana* [35] and *Spirulina platensis* [36] have been identified as both reducing and stabilizing agents for AgNPs. Castro et al. [37] demonstrated that galacturonic acid residues in the cell wall of *Spirulina platensis* can reduce Ag(I) to Ag(0) via hydroxyl groups. Carboxyl groups in alginate extracted from *Caulerpa racemosa* [38] and *Sargassum muticum* [39], a complex with Ag(I), are subsequently reduced to Ag(0) by hydroxyl groups [53]. Moreover, the polymeric alginate chains can bind Ag(I), thereby facilitating Ag(I) reduction and AgNPs nucleation. Meanwhile, sodium alginate binds strongly to AgNPs surfaces through hydroxyl and carboxyl groups, forming a negatively charged capping layer that effectively prevents nanoparticle aggregation [54]. Furthermore, hydroxyl, amino, carboxyl, and other functional groups present in compounds such as polyphenols, flavonoids, terpenoids, proteins, and lipids in the ethanolic extract of *Hormophysa triquetra* can reduce Ag(I) to Ag(0) [40].

Phycobiliproteins, including phycocyanin, phycoerythrin, allophycocyanin, and phycoerythrocyanin, represent an important class of algal components involved in AgNPs biosynthesis. Meanwhile, the functional groups within phycobiliproteins play key roles in AgNPs formation. The reducing capacity of components in *Porphyridium cruentum* extract decreases in the order: phycobiliprotein > lipids > proteins > polysaccharides. Hydroxyl, amino, carboxyl, and carbonyl groups have been identified as the key functional groups responsible for binding and reduction [41]. Furthermore, Ismail et al. [42] compared the similarities and differences in functional group utilization during AgNPs biosynthesis using crude phycobiliprotein extracts from *Spirulina platensis* and *Nostoc linckia*. The former involves multiple groups including hydroxyl, amino, and carboxyl groups, whereas the latter relies mainly on hydroxyl and amino groups.

#### 2.1.3. Bio-AgNPs Synthesized by Fungi

Fungi can synthesize AgNPs through both enzymatic and nonenzymatic reduction pathways involving multiple functional groups and reductases. In the filtrates of *Cladosporium halotolerans* [43] and *Ganoderma sessil* [44], alcoholic and phenolic hydroxyl groups have been identified as predominant reducing moieties. Proteins in the mycelial extract of *Ganoderma lucidum* mediate Ag(I) reduction through both carbonyl and hydroxyl groups [45]. In extracts of *Trichoderma harzianum* [44] and *Penicillium* sp. [46], amino groups in proteins play a major role in Ag(I) reduction. In addition to nonenzymatic pathways, enzymatic processes also play an important role in fungal-mediated AgNPs biosynthesis. For example, nitrate reductase purified from *Fusarium oxysporum* transfers electrons during the in vitro reduction of nitrate. This enzyme transfers a portion of electrons to the electron carrier 4-hydroxyquinoline, which then donates electrons to Ag(I), reducing it to Ag(0) [47]. Similar mechanisms have been observed in cell-free extracts of *Penicillium oxalicum*, *Neurospora intermedia* and *Gymnascella dankaliensis*, where NADH and NADH-dependent nitrate reductase jointly facilitate electron transfer to drive Ag(I) reduction [48,49,55].

After the formation of AgNPs, biomacromolecules can stabilize the nanoparticles by binding to their surfaces, thereby preventing aggregation. The negatively charged carboxyl groups in fungal filtrates bind AgNPs through electrostatic interactions, thereby enhancing the dispersibility of nanoparticles [46]. In *Ganoderma lucidum*, amino acid residues and proteins in the mycelial extract encapsulate AgNPs to form a protective capping layer, thereby preserving nanoparticle stability [45].

### 2.2. Bio-AuNPs Synthesis

Various microorganisms, including bacteria, algae, and fungi, synthesize AuNPs through diverse biochemical reduction pathways. Microbial biomolecules and reductase enzymes participate in Au(III) reduction, while proteins and polysaccharides contribute to nanoparticle stabilization. The following sections describe the biosynthesis of AuNPs in bacteria, algae, and fungi, respectively. The major mechanisms and key reactive components involved in the biosynthesis of AuNPs are summarized in Table 2.

#### 2.2.1. Bio-AuNPs Synthesized by Bacteria

Functional groups present in bacterial metabolites play a central role in the adsorption and reduction of Au(III). In *Marinobacter pelagius*, primary and secondary amines, carbonyls, and amino groups in secreted proteins reduce Au(III) to Au(0) [56]. Similarly, hydroxyl groups of phenolic and alcoholic compounds in the cell-free supernatants of *Lactiplantibacillus* sp. [58] and *Vibrio alginolyticus* [57] act as the major reductive moieties. In *Streptomyces* sp., amino and hydroxyl groups in proteins contribute to Au(III) reduction [59]. The ability of both heat-inactivated and viable *Pseudomonas aeruginosa* to synthesize AuNPs has been attributed to carboxyl groups on D-alanine and D-glutamate residues in peptidoglycan [60]. In addition, proteins can bind to AuNPs through free amine groups, cysteine, and lysine residues, forming protective capping layers that effectively prevent nanoparticle aggregation [56,58].

Among the secondary metabolites involved in microbial Au(III) reduction, only a few have been structurally and functionally characterized. Johnston et al. [61,81] reported that *Delftia acidovorans* produces delftibactin, a nonribosomal peptide that mediates extracellular Au(III) binding and converts it to insoluble gold species. This compound has subsequently been classified as a siderophore-like metallophore, referring to a class of metal-chelating molecules secreted by microorganisms. First, Hpa-1, a hydroxyphenylacetic acid-derived residue in delftibactin, and the two bidentate O,O-ligands of fhOrn-6 (N-formyl hydroxyornithine) facilitate the formation of square–planar Au(III) complexes. Subsequently, the N-formyl hydroxamate group of fhOrn-6 undergoes oxidative degradation and releases electrons. These electrons drive the stepwise reduction of Au(III) to Au(0) [82]. This process promotes the extracellular precipitation of colloidal and crystalline AuNPs and directly couples metal detoxification with biomineralization.

**Figure 3:**
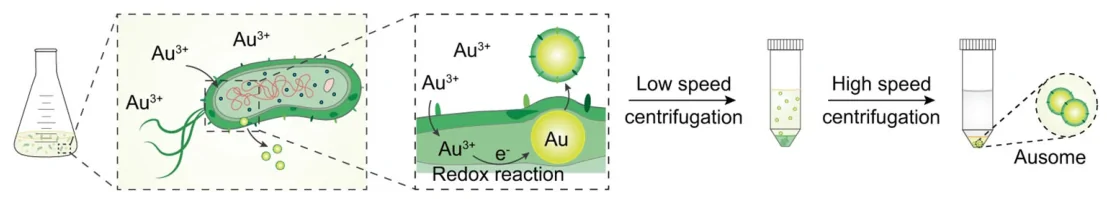
Schematic illustration of the biosynthesis and secretion of Ausome in *E. coli*. Reproduced from Qin et al. (2023) [64] with permission from the authors.

Bacteria can also mediate the biosynthesis of AuNPs through reductase enzymes. Nitrate reductase present in bacteria exhibits metal-reducing activity, providing an enzymatic pathway for AuNPs biosynthesis [83]. Sulfite reductase purified from *E. coli* directly reduces Au(III) to Au(0), using electrons donated by α-NADPH. Gelatin serves as a stabilizing agent that prevents aggregation of the resulting AuNPs [62]. In addition, *Azospirillum brasilense* secretes two phenol oxidases, manganese peroxidase and laccase, into the extracellular environment. Under acidic conditions, Fe(III) in manganese peroxidase is reduced to Fe(II). The reduced Fe(II) directly reduces Au(III) to Au(0). In the absence of H_2_O_2_, laccase interacts with O_2_ to generate H_2_O_2_. The generated H_2_O_2_ serves as a reducing agent to reduce Au(III) to Au(0) [63]. In *E. coli* [64], Au(III) is initially bound within EPS, where a small fraction of Au(III) is reduced to AuNPs by enzymes. As the Au(III) concentration increases, most Au(III) diffuses into the periplasmic space and undergoes enzymatic reduction. The accumulation of AuNPs in the periplasm triggers membrane budding and the subsequent release of membrane-coated vesicles into the extracellular environment. The resulting nanostructures, termed Ausomes, consist of an AuNPs core surrounded by immunostimulatory bacterial membrane components (Figure 3).

#### 2.2.2. Bio-AuNPs Synthesized by Algae

Algae are rich in biomacromolecules such as polysaccharides and proteins, and the functional groups within these biomolecules act as reducing agents during the synthesis of AuNPs. In the exudates of *Chondrus crispus*, *Gelidium corneum,* and *Porphyra linearis*, carbonyl and sulfate groups in proteins act as Au(III)-binding sites, while functional groups in polysaccharides donate electrons to Au(III) [65]. These polysaccharides can subsequently adsorb onto the surfaces of newly formed AuNPs through electrostatic interactions, forming a stabilizing layer that effectively prevents nanoparticle aggregation and precipitation [65]. In *Cystoseira baccata* and *Coelastrella thermophila,* hydroxyl groups in phenolic compounds, polysaccharides, and proteins are oxidized to carbonyl groups, thereby donating electrons for Au(III) reduction and the formation of AuNPs [66,67]. Extracts from *Chlorella sorokiniana* reduce Au(III) using amino groups, whereas the intracellular biosynthesis of AuNPs primarily depends on enzymatic catalysis [68]. In *Dunaliella salina* and *Spirulina* sp., functional groups in enzymes, proteins, phenolic compounds, vitamins, and flavonoids, including hydroxyl, carbonyl, and amine groups, interact with Au(III) and contribute to both the reduction and stabilization of AuNPs [69,70,84]. EPS from *Arthrospira platensis*, containing hydroxyl, amino, and carboxyl groups, has demonstrated the capacity to reduce Au(III) [71].

**Figure 4:**
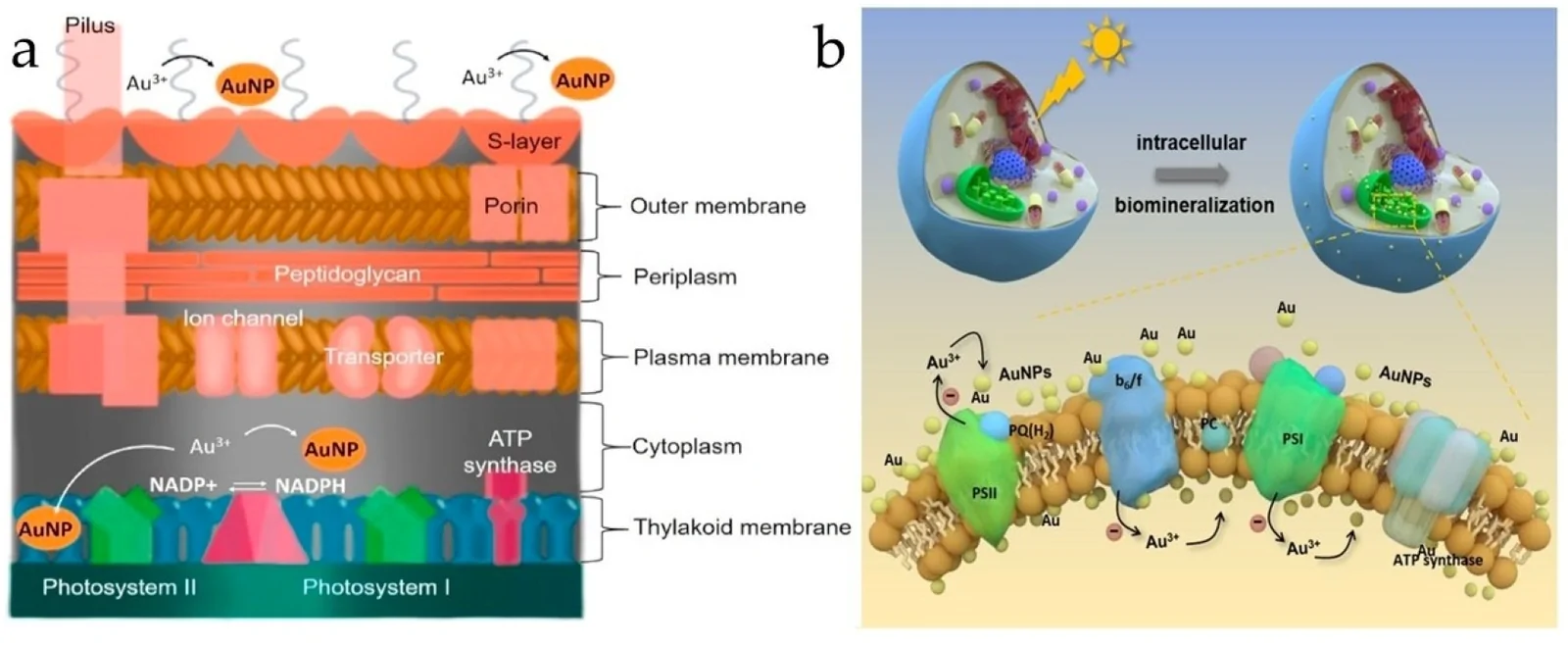
Schematic illustrations of Au(III) reduction mediated by photosynthetically derived electrons in photosynthetic microorganisms. (**a**) Schematic illustration of Au(III) reduction via photosynthetically derived electrons within *Synechocystis* sp. cells. Reprinted with permission from Elsevier. Reproduced from Liu et al. (2021) [72]. (**b**) Schematic illustration of Au(III) reduction via photosynthetically derived electrons within *Chlorella pyrenoidosa* cells. Reprinted with permission from Wiley. Reproduced from Zhu et al. (2023) [73].

Algae also achieve intracellular AuNPs synthesis by utilizing electrons from photosynthesis and respiration. Liu et al. [72] proposed that Au(III) is initially enriched on the surface of *Synechocystis* sp. through electrostatic interactions. Under anaerobic conditions, electrons generated from cellular respiration are transferred to Au(III) via EET. Under illumination, water photolysis at the thylakoid membranes generates electrons that are subsequently transferred to intracellular Au(III), resulting in the formation of AuNPs (Figure 4a). Zhu et al. [73] reported a similar mechanism, in which intracellular AuNPs formed in *Chlorella pyrenoidosa* are predominantly located around the thylakoid membrane (Figure 4b). This suggests that Au(III) entering the cytoplasm tends to associate with thylakoid membranes and acquires electrons through the photosynthetic electron transport chain (PSII → Cyt b6f → PSI). The generated AuNPs further enhance intracellular electron transfer efficiency and promote cellular photosynthetic activity. Compared with respiration-driven reduction, these two studies indicate that photosynthesis-derived electrons play a dominant role in AuNPs formation.

#### 2.2.3. Bio-AuNPs Synthesized by Fungi

The proteins, polysaccharides, and enzymes secreted by fungi can provide diverse reducing and stabilizing agents for green AuNPs synthesis. The contribution of secreted metabolites is largely dependent on their molecular weight: low-molecular-weight compounds primarily function as reducing agents, whereas high-molecular-weight proteins function as stabilizers [85,86,87,88]. For example, metabolites with molecular weights below 3 kDa, such as glucose and amino acids, are the major contributors to Au(III) reduction. In contrast, proteins larger than 3 kDa can strongly adsorb onto the surfaces of AuNPs through coordination or electrostatic interactions, thereby effectively preventing nanoparticle aggregation [86]. The electrons required for Au(III) reduction originate either from specific functional groups in biomolecules or from enzyme-mediated electron transfer processes.

Fungal redox enzymes can promote AuNPs synthesis by acting directly as reducing agents or by providing electrons generated from substrate oxidation [89]. During the reduction of Au(III) by *Myriococcum thermophilum*, electrons generated from cellobiose oxidation are sequentially transferred from the dehydrogenase domain to the cytochrome domain and ultimately transferred to Au(III), leading to the formation of AuNPs [74]. In *Glomerella cingulata*, glucose dehydrogenase facilitates AuNPs synthesis through endogenous reductants, including enzyme-bound glucose and amino acids, while supplementation with external glucose further accelerates the reaction [75]. In *Candida rugopelliculosa*, AuNPs synthesis proceeds through multiple coordinated steps: Au(III) is initially captured on the cell surface or internalized into the cell, where they accept electrons transferred from NADH-dependent reductases [90,91]. Subsequently, secreted extracellular polysaccharides and proteins facilitate further AuNPs formation, ensuring uniform particle distribution and long-term stability [76].

Fungal extracts or biomass can reduce Au(III) due to the abundant functional groups on polysaccharides and proteins. In *Flammulina velutipes* [78] and *Lentinula edodes* [77], hydroxyl groups in polysaccharides and amide groups in proteins act as the primary reducing agents. In extracts of *Agaricus bisporus*, hydroxyl, amino, and ether groups on amino acids and polysaccharides play critical roles in the reduction of Au(III) to Au(0) [79,92]. Similarly, amide, carboxyl, and hydroxyl groups present in proteins, lipids, and carbohydrates participate in the reduction process [93]. In *Alternaria chlamydospora*, Au(III) reduction has been attributed to hydroxyl and carboxyl groups in proteins, phenolic compounds, alcohols, and flavonoids [80].

### 2.3. Bio-PdNPs Synthesis

Bacteria, algae, and fungi employ diverse biological pathways for PdNPs biosynthesis, involving microbial functional groups, reductases, and metabolic activities for Pd(II) reduction. Biomolecules also contribute to nanoparticle stabilization by preventing aggregation. The major mechanisms and important reactive components involved in the biosynthesis of PdNPs are summarized in Table 3.

#### 2.3.1. Bio-PdNPs Synthesized by Bacteria

Bacterial synthesis of PdNPs is associated with its EET pathway, which includes both direct pathways (transfer through outer membrane c-type cytochrome protein) and indirect pathways (transfer through electron shuttles) [97]. In Gram-negative bacteria, electrons are transferred through a cytochrome network to outer-membrane c-type cytochromes, which act as reductases that reduce Pd(II) to Pd(0) [115]. For example, *Geobacter sulfurreducens* [94] can use acetate, formate, or hydrogen as the electron donor to synthesize PdNPs. In *Shewanella oneidensis*, Pd(II) ions are initially adsorbed onto the cell wall, after which electrons derived from anaerobic metabolism are delivered to the absorbed Pd(II) [95,96]. In contrast, Gram-positive bacteria lack the outer-membrane c-type cytochromes and therefore rely more on indirect electron transfer routes [116]. For example, *Staphylococcus saprophyticus* can synthesize PdNPs using formate as the electron donor [97]. Their study found that hydroxyl groups on the bacterial surface adsorb Pd(II), while riboflavin added to the medium functions as an electron shuttle [117], efficiently transporting intracellular electrons to the extracellular Pd(II). Notably, the resulting PdNPs can act as conductive centers, establishing a self-catalytic cycle that enhances electron transfer and accelerates Pd(II) reduction [97]. Furthermore, Bio-PdNPs formation may proceed through a hydrogen-mediated pathway. Hennebel et al. [98] demonstrated that PdNPs can initially be formed by accepting electrons from anaerobic respiration. Then, the PdNPs catalyze fermentation-derived hydrogen to generate active hydrogen, which acts as a powerful reducing agent to convert Pd(II) into PdNPs and deposited on the bacterial surface.

Beyond metabolically driven reduction, bacteria can also immobilize and reduce Pd(II) through functional groups present on the cell wall. In *Bacillus licheniformis*, Pd(II) coordinates with oxygen- and nitrogen-containing functional groups on the cell surface, after which polysaccharides are hydrolyzed to aldoses that serve as electron donors, reducing Pd(II) to PdNPs [99]. Tan et al. [100] reported that carboxyl and hydroxyl groups are the dominant Pd(II) adsorption sites on the *Providencia vermicola* cell wall, followed by phosphate and amine groups; the maximum biosorption capacity of the biomass reached 196.81 mg/g. The thiol group (-SH) is less abundant in the bacterial cell envelope; however, it can play a disproportionately important role in Pd(II) adsorption and reduction [118,119]. Supporting this, Tan et al. [101] demonstrated that *E. coli* engineered to display cysteine-enriched peptides exhibited enhanced Pd(II) adsorption; the maximum biosorption capacity of the biomass reached 163 mg/g, and chemical blocking of the thiol group markedly diminished Pd(II) binding capacity.

#### 2.3.2. Bio-PdNPs Synthesized by Algae

Algal extracts are rich in bioactive constituents, including polysaccharides, polyphenols, proteins, and polyols, which govern Pd(II) coordination, reduction, and nanoparticle stabilization via different functional groups. In *Sargassum cervicorne* extracts, alginate has been identified as the major reductant for Pd(II) [102]. Carboxyl groups in alginate coordinate with Pd(II) to form stable [Pd(Na-Alg)]^2+^ complexes, whereas hydroxyl groups act as electron donors to reduce Pd(II) [120]. Similarly, in extracts of *Botryococcus braunii* [103] and *Sargassum species* [104,105], polyphenols and polyols function as electron donors, with hydroxyl groups serving as the dominant reactive sites for Pd(II) reduction. In *Spirulina platensis* extracts [106], hydroxyl, carboxyl, ester and ether groups have been identified as the major functional moieties involved in Pd(II) reduction. In extracts of *Chlorella vulgaris* [107,108] and *Asterarcys* sp. [109], amide and hydroxyl groups in polyols play critical roles in Pd(II) reduction.

Moreover, biomolecules present in algal extracts impart both steric hindrance and electrostatic stabilization, thereby preventing the aggregation of high-surface-energy PdNPs. In *Sargassum* sp. [104], sulfated polysaccharides act as excellent capping agent, with their long-chain structures effectively encapsulating PdNPs and inhibiting aggregation. In *Botryococcus braunii* [103] and *Saragassum cervicorne* [102], the conversion of amino groups to amide groups has been detected during Pd(II) reduction, suggesting that proteins coordinate with the PdNPs surface via amide linkages.

#### 2.3.3. Bio-PdNPs Synthesized by Fungi

Fungi have also been explored for PdNPs biosynthesis, in which Pd(II) adsorption, reduction, and nanoparticle stabilization are mediated by functional groups on cell walls. Tan et al. [110] reported that yeast can adsorb Pd(II) and subsequently reduce it to Pd(0); furthermore, the addition of glutaraldehyde after Pd(II) adsorption can promote Pd(II) reduction without altering the chemical state of the resulting PdNPs. In *Neurospora crassa*, phosphate, amino, hydroxyl, and carboxyl functional groups on the cell wall contribute to Pd(II) adsorption, and PdNPs synthesis can be further promoted by treating Pd(II)-loaded mycelia with sodium formate [24]. Tarver et al. [111] demonstrated that *Phanerochaete chrysosporium* synthesizes PdNPs via coordination of Pd(II) to N and O atoms in amide groups, followed by reduction and nucleation mediated by reductive proteins. In addition to intact cells, fungal filtrates, which contain hydroxyl groups and functional moieties (e.g., phenolics and amides), have also been employed for PdNPs synthesis. Secondary metabolites present in filtrates of *Rhizopus* sp. [112], *S. cerevisiae* [113], and *Agaricus bisporus* [114] are participate both in the reduction and stabilization of PdNPs.

### 2.4. Bio-PtNPs Synthesis

The biosynthesis of PtNPs by bacteria, algae, and fungi involves reductase-mediated reduction, metabolic electron transfer, and biomolecule-assisted stabilization. These processes contribute to the conversion of Pt(IV) to Pt(0) and regulate nanoparticle formation. The following sections discuss the biosynthesis of PtNPs by bacteria, algae, and fungi in detail. The major mechanisms and important reactive components involved in the biosynthesis of PtNPs are summarized in Table 4.

#### 2.4.1. Bio-PtNPs Synthesized by Bacteria

Reductive enzymes, located both intracellularly and in the periplasmic space of bacteria, are the primary contributors to PtNPs synthesis. Intracellular reduction is generally regarded as a detoxification strategy that converts toxic Pt(IV) ions into relatively inert PtNPs. For example, Pt(IV) can enter *Acinetobacter calcoaceticus* cells via active transport or passive diffusion and is subsequently reduced by intracellular reductases [121]. In *Bacillus* sp., nitrate reductases and NADH-dependent reductases function as both reducing and capping agents, facilitating Pt(IV) reduction and stabilizing the resulting PtNPs [122]. Pt(IV) reduction has been observed both intracellularly and extracellularly in viable *Cupriavidus metallidurans* cells, whereas dead cells only adsorb Pt(IV) without forming PtNPs [137]. Intracellular detoxification of Pt(IV) can damage organelles, causing membrane rupture and cell death. Consequently, bacteria often localize Pt(IV) reduction in the periplasmic space or extracellular environment rather than the cytoplasm. In *Desulfovibrio vulgaris*, periplasmic hydrogenases are primarily responsible for Pt(IV) reduction. Electrons generated from H_2_ oxidation by these hydrogenases are transferred to Pt(IV), forming PtNPs that are subsequently released and deposited on the cell surface, where they catalyze further Pt(IV) reduction [123]. A stepwise mechanism mediated by two hydrogenases has also been proposed for sulfate-reducing bacteria: a cytoplasmic hydrogenase reduces Pt(IV) to Pt(II), which is then transported to the periplasm for further reduction to PtNPs [124]. In addition to hydrogenases, periplasmic formate dehydrogenases (FDHs) can drive PtNPs formation. Acidophilic Fe(III)-reducing bacteria can utilize FDHs to decompose formate and release H_2_, which reduces Pt(IV) to form initial PtNPs. These initially formed PtNPs act as catalysts, accelerating formate decomposition and promoting further PtNPs growth [125].

Functional groups in bacterial cell walls and metabolites can adsorb and reduce Pt(IV). In *E. coli*, amino, hydroxyl, carboxyl, sulfhydryl, and phosphate groups act as primary adsorption sites and interact with surface proteins to form stable PtNPs. The engineered *E. coli* strain exhibited a maximum adsorption capacity of 121.84 mg/g for Pt(IV), and achieved a 100% removal efficiency of Pt(IV) from wastewater [126,127]. In *Bacillus megaterium*, reducing sugars generated from hydrolysis of polysaccharide reduce Pt(IV), during which free aldehyde groups are oxidized to carboxyl groups [128]. This reduction proceeds stepwise, with rapid conversion of Pt(IV) to Pt(II) followed by slower reduction to Pt(0).

#### 2.4.2. Bio-PtNPs Synthesized by Algae

Algal extracts contain diverse biomolecules that act as both reducing and stabilizing agents during PtNPs biosynthesis. Biomolecules, such as proteins and polysaccharides in algae, can coordinate with PtNPs through amino and carboxyl groups, forming a stable layer that prevents aggregation. In algae extracts [138,139,140], hydroxyl and amino groups in biomolecules serve as key reductants driving the conversion of Pt(IV) to nanoparticles. After reduction, C=C bonds, esters, aromatic groups, and isothiocyanates cap and stabilize PtNPs via coordination and steric hindrance. In *Sargassum polyphyllum* extract, ethyl 6,9,12-hexadecatrienoate is considered the primary reductant, whereas methyl 2-methoxytricosanoate, isopropyl(dimethyl)silyl palmitate, oleoyl chloride, cinnamyl linoleate, and linoleoyl chloride contribute to the encapsulation of PtNPs [129]. In *Halymenia dilatata* extract, phenolic hydroxyl groups in polyphenols and flavonoids act as reducing agents for Pt(IV) reduction [130].

In some cyanobacteria, nitrogenase provides reducing power that can be redirected to reduce Pt(IV) to Pt(0). For example, *Calothrix pulvinata* and *Anabaena flos-aquae* possess nitrogenase that normally reduces atmospheric N_2_ to NH_3_ using electrons as a reducing power [131,141]. In the presence of Pt(IV), these electrons can be diverted to reduce Pt(IV) to Pt(0). The reduction occurs in heterocysts and vegetative cells, after which PtNPs are released into the medium through intercellular canal-like cytoplasmic connections.

#### 2.4.3. Bio-PtNPs Synthesized by Fungi

Fungi serve as versatile ‘nanofactories’ for PtNPs synthesis, utilizing both secreted metabolites and intracellular enzymes [142]. For example, *Penicillium canescens* secretes secondary metabolites such as oxalic and citric acids, which are capable of reducing adsorbed Pt(IV) ions [132]. Proteins and peptides within secondary metabolites in *Streptomyces* sp. contain numerous amino groups, which act as effective reductants for Pt(IV) reduction [133]. Meanwhile, functional groups such as amino, carboxyl, and hydroxyl moieties in the secondary metabolites bind firmly to the surfaces of nascent PtNPs, forming a dense organic protective layer that prevents aggregation [143]. Enzymes and quinones present in *Alternaria alternata* filtrates reduce Pt(IV) to Pt(0), while secreted proteins function as capping agents that stabilize the newly formed PtNPs [134].

The mechanisms underlying PtNPs synthesis in *Fusarium oxysporum* have been extensively investigated. Syed et al. [136] suggested that hydroxyl-containing secretions from *F. oxysporum* act as reducing agents for Pt(IV), whereas proteins stabilize the resulting PtNPs through free amino groups or cysteine residues [135]. Extracellular reductive enzymes, potentially hydrogenases, can directly reduce Pt(IV) to Pt(0) in culture, thereby initiating the nucleation and growth of PtNPs [144]. Notably, purified hydrogenase from *F. oxysporum* extracts exhibits a Pt(IV) reduction efficiency of up to 90% [145]. The hydrogenase in *F. oxysporum* contains a network of hydrophobic channels connecting the active site to the molecular surface, which facilitates metal ion transport [19]. Although the channel diameters (approximately 0.45–0.60 nm) are too small to accommodate octahedral H_2_PtCl_6_, they are accessible to the smaller planar PtCl_2_ species. Under alkaline conditions (pH 9) and elevated temperature (65 °C), H_2_PtCl_6_ is initially reduced via a passive two-electron transfer at a remote surface site. The resulting Pt(II) species subsequently migrates through the channel network to the active site, where it undergoes a second, slower two-electron reduction to Pt(0).

## 3. Applications of Bio-PMNPs

Compared with nanoparticles synthesized by physical and chemical methods, Bio-PMNPs generally exhibit superior biocompatibility, stability, dispersibility, and catalytic performance. Consequently, Bio-PMNPs have found widespread applications in biomedicine, environmental remediation, catalysis, and energy conversion. The following sections describe the major applications of Bio-AgNPs, Bio-AuNPs, Bio-PdNPs, and Bio-PtNPs. In addition, the main applications of Bio-PMNPs are summarized in Table 5.

### 3.1. Applications of Bio-AgNPs

Compared with chemically synthesized AgNPs, Bio-AgNPs are often capped by biomolecules, which enhance their stability and biocompatibility. Consequently, Bio-AgNPs have attracted increasing attention for applications in antimicrobial therapy, cancer treatment, antioxidant systems, and environmental catalysis.

Bio-AgNPs exhibit strong antibacterial activity, particularly against multidrug-resistant bacteria, highlighting their promising potential as novel antibacterial drugs. For example, *Paenarthrobacter nicotinovorans* can synthesize Bio-AgNPs that significantly disrupt cell wall integrity, resulting in damaged and porous cell surfaces and ultimately leading to bacterial death [31]. Bio-AgNPs synthesized by *Bacillus subtilis* [146] and *Microcoleus* sp. [147] exhibit broad-spectrum antibacterial activity against multiple Gram-negative bacteria (e.g., *Escherichia coli*, *Pseudomonas aeruginosa*, *Proteus vulgaris*) and Gram-positive bacteria (e.g., *Staphylococcus aureus*, *Bacillus subtilis*, *Corynebacterium* sp.). In contrast, Bio-AgNPs synthesized by *Lactobacillus plantarum* show stronger inhibitory effects against Gram-negative bacteria than Gram-positive strains [148]. The antibacterial activity of Bio-AgNPs may be attributed to several mechanisms, including (1) Ag(I) ions released from AgNPs interact with bacterial cell surfaces, particularly binding to functional groups in proteins, resulting in protein deactivation. (2) Direct physical contact between AgNPs and the cell wall can facilitate nanoparticle penetration into the cells, which may exert toxic effects on intracellular biomolecules.

Bio-AgNPs can modulate quorum sensing (QS), thereby influencing bacterial virulence traits and biofilm formation. However, their effects on QS regulation differ with the inhibitory concentration and the target strain. Bio-AgNPs biosynthesized by the probiotic *Lactobacillus rhamnosus* (AgNPs-LR) exhibit not only antibacterial activity but also inhibitory effects on QS-mediated virulence traits and biofilm formation in Gram-negative pathogens [149]. Specifically, AgNPs-LR markedly inhibited the production of the QS-regulated pigments violacein in *Chromobacterium violaceum* and prodigiosin in *Serratia marcescens*, with maximum inhibition rates of 75.49% and 71.28%, respectively. In Pseudomonas aeruginosa, AgNPs-LR dose-dependently reduced the production of several QS-controlled virulence factors, including pyocyanin, pyoverdine, LasA protease, LasB elastase, and rhamnolipids. In addition, AgNPs-LR decreased bacterial aggregation, EPS production, and biofilm formation. In contrast, Bio-AgNPs synthesized using the cell-free filtrate of *Fusarium oxysporum* exhibited a distinct pattern of QS modulation at sub-inhibitory concentrations [150]. These Bio-AgNPs did not significantly affect the growth of *P. aeruginosa* PAO1 or PA14, whereas the expression of several QS-related genes, including lasI, lasR, pqsA, and mvfR, was upregulated. However, the motility, rhamnolipid production, and elastase activity of the PAO1 strain were reduced, whereas the PA14 strain exhibited the opposite trend. These findings indicate that transcriptional changes in QS-related genes do not necessarily correlate with the resulting virulence phenotypes. Therefore, the effects of Bio-AgNPs on QS are not limited to simple inhibition or activation. Instead, they may alter bacterial pathogenic behaviors by disrupting QS network homeostasis and its downstream regulatory pathways.

Bio-AgNPs show promising potential in cancer therapy, exhibiting effective anticancer activity with fewer side effects. Bio-AgNPs can induce tumor cell apoptosis or activate immune responses to eliminate cancer cells [151]. For instance, Bio-AgNPs synthesized by *Spirulina platensis* have been shown to trigger apoptosis in human hepatocellular carcinoma (HepG2) cells, likely through interactions with intracellular DNA or proteins [36]. In addition, Bio-AgNPs synthesized by *Aspergillus* sp. promote the release of cytokines, including IL-1, IL-1β, and TNF-α, thereby activating immune responses and inducing tumor cell apoptosis [152]. Bio-AgNPs produced by *Lactobacillus paracasei* significantly reduce the viability of CAL-51 and MCF-7 breast cancer cells in a time- and concentration-dependent manner [42].

Bio-AgNPs exhibit significant antioxidant activity, supporting their potential application in reactive oxygen species scavenging. During wound healing and inflammation control, Bio-AgNPs can effectively scavenge reactive oxygen species at the wound site while simultaneously inhibiting bacterial growth [153]. Bio-AgNPs synthesized by *Bacillus cereus* [30], *Spirulina platensis* [36] and *Nostoc linckia* [42] demonstrated notable free-radical scavenging activity. The antioxidant activity of Bio-AgNPs synthesized by *Lactobacillus plantarum* is attributed to their ability to donate electrons to DPPH radicals, thereby neutralizing their unpaired electrons [148]. In addition, AgNPs themselves can undergo oxidation in the presence of oxygen, thereby neutralizing free radicals. However, this process may affect the activity and stability of Bio-AgNPs [154].

Bio-AgNPs show excellent performance in both heterogeneous and homogeneous catalysis, enabling broad application in organic pollutant degradation and environmental remediation [155]. For example, p-nitrophenol (4-NP) is a toxic nitroaromatic compound widely used in the synthesis of pharmaceuticals, dyes, and pesticides, yet it is persistent and difficult to degrade naturally [156]. Bio-AgNPs synthesized by *Cylindrocladium floridanum* can efficiently catalyze the reduction of 4-NP to *p*-aminophenol in the presence of NaBH_4_. This rapid and efficient transformation is attributed to the ability of AgNPs to lower the kinetic barrier for electron transfer from NaBH_4_ to the nitro group [110,157]. Notably, Bio-AgNPs synthesized by *Bacillus paralleleniformis* effectively degrade malachite green owing to their high specific surface area [26]. In addition, Bio-AgNPs from *Letendraea* sp. catalyze the degradation of methyl orange, rhodamine B, and methylene blue within 20 min [158].

### 3.2. Applications of Bio-AuNPs

Bio-AuNPs possess excellent catalytic activity, outstanding biocompatibility, strong photothermal effects, and enhanced electron transfer capability, making them highly promising functional nanomaterials for applications in environmental remediation, biomedicine, and energy conversion.

In the field of environmental remediation, Bio-AuNPs play a critical role in removing heavy metal ions and organic pollutants by acting as catalysts or high-capacity adsorbents. Tan et al. [1] reported that Bio-AuNPs on carbonized *Saccharomyces cerevisiae* can efficiently adsorb and reduce toxic Cr(VI) to less toxic Cr(III), exhibiting removal efficiencies significantly higher than that of conventional activated carbon. Bio-AuNPs accelerate the transfer of electrons from the cathodic biofilm to the Cr(VI) ions by reducing the electron transfer impedance, enhancing the electron conductivity at the electrode surface [159]. Additionally, Bio-AuNPs synthesized by *Rhizopus oryzae* are capable of removing pesticides via chemisorption while simultaneously inactivating *E. coli* within a short time, highlighting their potential in wastewater treatment [160]. In the presence of NaBH_4_, Bio-AuNPs produced by *Penicillium rubens* can catalyze electron transfer to effectively degrade 4-nitrophenol, phenol red, congo red, bromothymol blue, and methylene blue [161].

Their unique biocompatibility and photothermal properties provide Bio-AuNPs with great potential in anticancer therapy. The antitumor mechanisms of AuNPs are multifaceted, including the induction of apoptosis, the generation of oxidative stress, and performance as photothermal agents. In photothermal therapy, Bio-AuNPs are utilized to induce tumor cell apoptosis by generating localized hyperthermia under laser irradiation [162]. Specifically, Bio-AuNPs synthesized by *Streptomyces albogriseolus* can activate caspase cascades and generate excessive reactive oxygen species by interacting with cellular DNA and proteins, thereby inducing apoptosis in hepatic cancer cells [163]. Moreover, Bio-AuNPs produced by *Phormidesmis communis* exhibit excellent biocompatibility with normal cells while demonstrating significant cytotoxicity toward osteosarcoma cells by inducing apoptosis or cell cycle arrest [164].

In the context of energy conversion, Bio-AuNPs can markedly enhance photoelectric conversion efficiency owing to their high electrical conductivity and localized surface plasmon resonance (LSPR) effects. Mechanistically, Bio-AuNPs improve light harvesting by acting as electron bridges and by lowering interfacial charge-transfer resistance. For example, Bio-AuNPs synthesized by *Synechocystis* sp. significantly enhance the light-harvesting capability of cyanobacteria [72]. In bio-photovoltaic systems, AuNPs function as highly efficient ‘electron conduits’, electrically coupling intracellular components with external electrodes, thereby facilitating EET and increasing overall electrical output [72]. Furthermore, Zhu et al. [73] developed a photosynthesis-mediated biomineralization strategy to generate intracellular AuNPs in *Chlorella pyrenoidosa* cells, in which photosynthesis-driven reduction of Au(III) to AuNPs occurs around the thylakoid membrane. Notably, electrons generated via the LSPR excitation of AuNPs substantially enhance hypoxic photosynthesis, promoting photoelectron generation and transport along the photosynthetic electron-transfer chain and ultimately boosting hydrogen production under sunlight.

### 3.3. Applications of Bio-PdNPs

Bio-PdNPs exhibit exceptional dispersibility, stability, and biocompatibility due to the unique capping effect of biomolecules. These outstanding properties allow Bio-PdNPs to have remarkable potential and prospects in environmental remediation, organic synthesis, microbial fuel cells, chemical sensors, and antibacterial applications.

Bio-PdNPs have emerged as effective catalysts for environmental remediation. For example, Bio-PdNPs synthesized by *S. cerevisiae* and *Neurospora crassa* can effectively catalyze the reduction of highly toxic Cr(VI) to Cr(III), with catalytic efficiency strongly dependent on the Pd(0) content [1,24]. Bio-PdNPs produced by *Staphylococcus saprophyticus* exhibit high catalytic activity toward the degradation of methyl orange [97]. Similarly, Bio-PdNPs synthesized by *Sargassum cervicorne* act as electron transfer mediators to catalyze the reduction of azo dyes [102]. The removal mechanism of organic pollutants involves adsorption of hydrogen generated from sodium borohydride onto Bio-PdNPs, followed by cleavage of chromophoric azo bonds in dye molecules, yielding colorless aromatic amines [102]. Bio-PdNPs also exhibit considerable potential for the degradation of recalcitrant halogenated organic pollutants. For example, Bio-PdNPs synthesized by *Enterococcus faecium* act as catalysts that dissociate hydrogen to produce highly active hydrogen [98]. The active hydrogen adsorbed on the surface of Bio-PdNPs enhances their catalytic dehalogenation capacity toward diatrizoate and trichloroethylene, thereby facilitating their degradation.

In organic synthesis, Bio-PdNPs act as heterogeneous catalysts that enable a wide range of bond-forming reactions with high efficiency, selectivity, and sustainability [165]. For example, Bio-PdNPs synthesized by *Asterarcys* sp. have been applied in Mizoroki–Heck and Suzuki–Miyaura reactions, achieving high yields in ethyl cinnamate and 1,1′-biphenyl production [109]. Bio-PdNPs are also effective catalysts for the Heck and Buchwald–Hartwig reactions, the latter being a key transformation for constructing C-N bonds in pharmaceutical molecules [111,166]. For instance, Bio-PdNPs synthesized by *Chlorella vulgaris* have been employed to catalyze the formation of N-aryl piperazine compounds with significant pharmacological activities [107]. Bio-PdNPs produced by *Botryococcus braunii* act as efficient catalysts to allow the one-pot synthesis of various 2-substituted quinoline derivatives [103].

Bio-PdNPs have been applied in microbial fuel cells, chemical sensing, and antibacterial applications. For instance, Bio-PdNPs synthesized by *Citrobacter* sp. have been used as anodic electrocatalysts in microbial fuel cells, significantly enhancing power output [25]. This improvement is attributed to the abundant catalytically active sites provided by Bio-PdNPs immobilized on the anode, which promote formate oxidation and accelerate electron transfer [167]. Moreover, Bio-PdNPs synthesized by *Sargassum* sp. have been used to modify electrode surfaces in enzyme-free electrochemical sensors, enabling rapid and highly selective detection of H_2_O_2_ with low detection limits [104]. This performance arises from the ability of surface-immobilized Bio-PdNPs to catalyze the electrochemical reduction of H_2_O_2_ and effectively transduce the reaction into an electrical signal. In addition, Bio-PdNPs exhibit strong antibacterial activity. They show excellent photothermal bactericidal effects against *Staphylococcus aureus* and *E. coli* [168]. Bio-PdNPs synthesized by *Agaricus bisporus* also demonstrate broad-spectrum antimicrobial activity against both Gram-positive and Gram-negative bacteria [114].

### 3.4. Applications of Bio-PtNPs

Compared with conventionally synthesized PtNPs, Bio-PtNPs exhibit superior catalytic activity, targeting capability, and biocompatibility, making them promising materials for antitumor therapy, antimicrobial treatment, and pollutant removal.

In antitumor applications, Bio-PtNPs can selectively inhibit cancer cell proliferation and exhibit enhanced efficacy when combined with other therapies. Their anticancer activity is associated with mitochondrial dysfunction, oxidative stress, DNA damage, increased nitric oxide production, and elevated intracellular Ca^2+^ influx [129]. Bio-PtNPs can also activate p53-dependent apoptotic pathways and induce gene expression changes related to programmed cell death [129]. Notably, they show selective cytotoxicity toward human breast cancer cells (MCF-7) while exhibiting minimal toxicity to normal cells [122,129,130,133]. To further improve in vivo antitumor efficacy, bacterial membrane fragments derived from *Shewanella oneidensis* have been used to load Bio-PtNPs (PtMFs) [169]. During radiotherapy, PtMFs enhance radiation-induced DNA damage and tumor oxygenation, thereby alleviating hypoxia-induced radioresistance and remodeling the immunosuppressive tumor microenvironment.

Bio-PtNPs exhibit broad-spectrum antimicrobial activity and strong free radical-scavenging ability. They show significant inhibitory effects against Gram-positive bacteria (e.g., *Staphylococcus aureus*), Gram-negative bacteria (e.g., *E. coli*, *Acinetobacter baumannii*, *Klebsiella pneumoniae*, and *Pseudomonas aeruginosa*), and fungi (e.g., *Aspergillus niger*) [133,135,139,140,170]. PtNPs produced by *Halymenia dilatata* can disrupt cell membrane integrity, leading to intracellular protein leakage and bacterial death [130]. The antimicrobial efficacy of PtNPs can be further enhanced by incorporation into polyvinylpyrrolidone (PVP), forming PVP/PtNPs nanocomposites that produce larger inhibition zones against seven pathogenic bacteria than either PtNPs or PVP alone [138]. These nanocomposites also exhibit strong antioxidant activity by scavenging superoxide anions, nitric oxide, and hydroxyl radicals [138]. Similarly, Bio-PtNPs synthesized by *Halymenia dilatata* and *Caulerpa sertularioides* show notable DPPH radical scavenging activities of up to 59.72% and 74%, respectively [130,140].

In environmental remediation, Bio-PtNPs serve as efficient and reusable catalysts for degrading persistent organic pollutants and detoxifying heavy metals. They can effectively remove refractory pharmaceutical contaminants such as sulfamethoxazole, ciprofloxacin, and endocrine-disrupting compounds [171]. Notably, Bio-PtNPs can convert β-estradiol in aquatic systems into less toxic derivatives, reducing the risk of secondary pollution [171]. These nanoparticles also exhibit good stability and reusability in heterogeneous systems, retaining 69–86% of sulfamethoxazole removal efficiency after three cycles [171]. In heavy metal remediation, Bio-PtNPs synthesized by *Acidocella aromatica* and *Acidiphilium cryptum* efficiently catalyze the reduction of highly toxic Cr(VI), with catalytic performance inversely correlated with nanoparticle size [125].

## 4. Other Bio-PMNPs Synthesis and Applications

Although microbial synthesis offers a sustainable and green strategy for producing PMNPs, the biosynthesis of rhodium (Rh) and ruthenium (Ru) nanoparticles remains relatively underexplored. To our knowledge, Ngwenya et al. [172] first reported the bioreductive recovery of rhodium using sulfate-reducing bacteria (SRB) under a hydrogen atmosphere. In this process, Rh(III) ions were initially adsorbed onto the negatively charged cell surface and subsequently reduced intracellularly by hydrogenases, leading to the formation of RhNPs dispersed intracellularly. Over time, RhNPs accumulated in the periplasmic space and extracellular medium, suggesting active export through energy-dependent efflux. Tamaoki et al. [173] reported that *Shewanella algae* could take up soluble Rh(III) and reduce it intracellularly using sodium lactate as the electron donor. Srivastava et al. [174] demonstrated extracellular biosynthesis of RhNPs and RuNPs by *Pseudomonas aeruginosa*, likely mediated by amino groups in secreted organic compounds. Gómez Bolívar et al. [175] synthesized RuNPs and Pd/Ru bimetallic nanoparticles using *Escherichia coli*. In this system, Pd(II) was preferentially reduced to PdNPs, which subsequently catalyzed Ru(III) reduction, leading to the formation of Ru-core/Pd-shell nanoparticles. Bio-Pd/Ru bimetallic nanoparticles produced by *E. coli* have been demonstrated as efficient catalysts for the conversion of 5-hydroxymethylfurfural into the liquid fuel precursor 2,5-dimethylfuran [175].

## 5. Conclusions and Future Scope

Microbial synthesis of PMNPs has emerged as a green nanotechnology for the production of functional nanomaterials in an environmentally friendly, sustainable, and biocompatible manner. Bacteria, fungi, and algae can mediate the reduction of precious metal ions, forming AgNPs, AuNPs, PdNPs, PtNPs, RhNPs, and RuNPs. Functional groups on the bacterial surface, including hydroxyl, amino, carboxyl, thiol, and carbonyl groups, play critical roles by coordinating metal ions, facilitating electron transfer, and stabilizing the growing nanoparticles. In addition, various biomolecules participate in the reduction of precious metal ions. Reductive enzymes, such as hydrogenases and nitrate reductases, mediate electron transfer to metal ions. Small molecular metabolites, including organic acids, polyphenols, and other secondary metabolites, can also function as reducing agents. In addition, the capping layer formed by biomolecules on the nanoparticle surface is an important factor affecting the stability of PMNPs. The composition and structure of the capping layer vary among different biological sources. Capping layers rich in proteins and polysaccharides generally improve colloidal stability by providing both steric hindrance and electrostatic repulsion, thereby reducing nanoparticle aggregation. In contrast, capping layers mainly composed of small-molecule metabolites usually provide weaker stabilization. Therefore, the composition of the capping layer is considered an important factor influencing the size uniformity, dispersibility, and long-term stability of PMNPs. However, quantitative studies on the relationship between capping layer composition and PMNPs stability remain limited.

Bio-PMNPs have been widely investigated in biomedicine, environmental remediation, catalysis, and energy conversion owing to their excellent biocompatibility, stability, and catalytic performance. Bio-AgNPs exhibit antibacterial, antioxidant, and catalytic activities and have been applied in antimicrobial therapy, cancer treatment, and organic pollutant degradation. Bio-AuNPs possess photothermal properties, electron transfer capability, and catalytic performance, showing potential applications in environmental remediation, anticancer therapy, and photoelectric conversion. Bio-PdNPs can function as heterogeneous catalysts in organic synthesis, pollutant degradation, electrocatalysis, and chemical sensing, while also exhibiting antibacterial activity. Bio-PtNPs exhibit cytotoxic effects against various cancer cells and possess antibacterial, antioxidant, and catalytic activities for organic pollutant degradation and heavy metal reduction. Overall, microbial synthesis provides a feasible green approach for the preparation of PMNPs and facilitates their application in medicine, catalysis, energy conversion, and environmental remediation.

Despite substantial progress in microbial PMNPs synthesis, several key knowledge gaps continue to limit both mechanistic understanding and practical application. Current research remains largely phenomenological, with limited resolution of the molecular mechanisms underlying PMNPs biosynthesis. In extracellular systems, the metabolites directly responsible for precious metal reduction have yet to be identified, and the specific molecular species mediating electron transfer and metal transformation remain poorly characterized. Similarly, the biological regulation of nanoparticle nucleation, growth, and maturation is still not well understood, restricting the precise control of particle size, morphology, and crystal structure. Elucidation of the metabolic pathways, electron transfer networks, and regulatory mechanisms involved in PMNPs formation will be essential for the rational engineering of microbial nanofactories. In parallel, major challenges associated with nanoparticle productivity, process stability, and economic feasibility continue to impede industrial-scale implementation. Overcoming these limitations represents a prerequisite for the translation of microbial PMNPs synthesis into scalable and commercially viable technologies.

Besides the lack of mechanistic research, reproducibility remains a significant challenge for microbial synthesis of PMNPs. Because the formation of Bio-PMNPs depends on the metabolic activity of living microorganisms, variations in microbial species, culture conditions, pH, temperature, and metal precursor concentration can result in significant differences in nanoparticle size, morphology, and surface properties. Therefore, establishing standardized cultivation protocols and synthesis procedures is essential for improving the reproducibility and reliability of Bio-PMNPs production.

Most studies on Bio-PMNPs are still limited to the laboratory scale. Efficient recovery and purification of Bio-PMNPs from complex microbial systems remain major challenges for their practical application. For intracellularly synthesized Bio-PMNPs, recovery usually requires cell disruption by ultrasonication, mechanical homogenization, or enzymatic lysis. The nanoparticles are then separated from cell debris by centrifugation, filtration, or density gradient separation. In contrast, extracellularly synthesized nanoparticles are easier to recover from cell-free supernatants. They can be collected by centrifugation, ultrafiltration, dialysis, and repeated washing. However, complete removal of microbial biomolecules remains difficult because proteins, polysaccharides, and other metabolites are strongly associated with the nanoparticle surface through adsorption or coordination interactions. These biomolecules also contribute to nanoparticle stability, making purification more challenging.

Insufficient purification of Bio-PMNPs may also raise concerns regarding biosafety evaluation. Residual microbial components, including bacterial endotoxins, fungal spores, cell debris, and extracellular toxic metabolites, may interfere with biological applications and affect toxicity assessment. Therefore, it is important to distinguish whether the biological activity and toxic effects of Bio-PMNPs originate from the nanoparticles themselves or from residual microbial components. This issue is particularly important for biomedical applications. Incompletely purified Bio-PMNPs may induce unintended immune responses, inflammation, or cytotoxicity. Therefore, effective purification strategies and rigorous quality control are essential to ensure the biosafety, reproducibility, and future application of Bio-PMNPs.

These challenges also limit the translation of Bio-PMNPs from laboratory research to practical applications and commercialization. Although Bio-PMNPs have shown great potential in catalysis, biomedicine, and environmental remediation, their commercial use remains limited compared with PMNPs produced by conventional physicochemical methods. Among them, Bio-AgNPs are the most extensively studied because of their strong antibacterial activity and promising applications in antimicrobial materials, wound dressings, and other biomedical fields. Bio-AuNPs have attracted considerable attention for biosensing and photothermal therapy because of their unique optical properties. In addition, Bio-PdNPs have shown great potential in catalytic reactions and the degradation of environmental pollutants. Despite these advances, most studies on Bio-PMNPs are still at the laboratory scale. Their industrial translation is restricted by several factors, including the high cost of downstream purification, poor batch-to-batch reproducibility, the lack of standardized production processes, and incomplete safety evaluation and regulatory frameworks. Future efforts should focus on optimizing microbial cultivation systems, developing efficient purification technologies, and establishing strict quality evaluation criteria. These advances will facilitate the translation of Bio-PMNPs from laboratory research to practical applications.

Future studies should integrate synthetic biology and genetic engineering approaches to better control microbial nanoparticle synthesis. Strategies such as gene knockout, gene insertion, surface display systems, or overexpression of metal-binding proteins may enhance the selective adsorption of precious metal ions and improve nanoparticle formation efficiency. Identifying key reductases, metabolic intermediates, and electron-transfer pathways will also help reveal the mechanisms controlling nanoparticle nucleation and structural evolution. In industrial applications, the construction of biologically derived substrate systems using agricultural waste, industrial fermentation by-products, and microbial cells can reduce raw material costs. By regulating key parameters such as temperature, pH, and substrate concentration, the size distribution and morphological uniformity of nanoparticles can be improved within a certain range. The integration of separation, purification, and drying processes can enhance product recovery and purity while improving overall process efficiency. The biosynthesis of PMNPs can be coupled with precious metal-containing waste streams to enable resource recovery and reuse. To address issues related to stability, selectivity, and batch-to-batch consistency, surface modification and immobilization on porous supports are commonly employed to improve dispersion stability and reusability, while bimetallic structures and compositional tuning may enhance catalytic selectivity. In terms of process control, strain screening or engineering, cell-free systems, and online monitoring coupled with feedback regulation can improve process controllability and reproducibility. Biosafety assessment typically relies on standardized toxicological evaluation methods, and biodegradable coating materials are often applied to reduce potential bio-related risks.

Overall, advances in microbiology, nanotechnology, and synthetic biology are expected to transform microbial synthesis into a controllable and scalable platform for producing PMNPs with broad applications in catalysis, environmental remediation, energy conversion, and biomedicine.

## Figures and Tables

**Table 1:** Summary of Bio-AgNPs synthesized by microorganisms.

Microorganism	Species	Main Mechanism	Key Molecules/ Functional Groups	References
Bacteria	*Bacillus pumilus*, *Bacillus paralicheniformis*, *Sphingomonas paucimobilis*, *Bacillus lentus*	Functional groups in the cell-free extract mediate the synthesis and stabilization of AgNPs	Amino, carbonyl groups	[26,27]
Bacteria	*Bacillus megaterium*, *Bacillus subtilis*, *Bacillus cereus*, *Paenarthrobacter* *nicotinovorans*	Biomolecules within the bacterial extract mediate the reduction process of AgNPs	Proteins, enzymes, peptides, polysaccharides, organic acids	[28,29,30,31]
Bacteria	*Bacillus clausii*, *Bacillus subtilis*	Enzymatic reduction of AgNPs	Nitrate reductase, NADH	[32,33]
Bacteria	*Pantoea* sp.	AgNPs are formed through multiple regulatory processes, including biosorption, intracellular transport, bioreduction, and cellular detoxification	Hemiacetal groups, thiol group, nitrate reductase, nitric oxide reductase, CopA, CueO, arsenate reductase et al.	[34]
Algae	*Ulva armoricana*, *Spirulina platensis*	Functional groups of biomolecules in the cell extract adsorb and reduce AgNPs	Sulfated polysaccharides	[35,36]
Algae	*Spirulina platensis*	Functional groups on the cell wall adsorb and reduce AgNPs	Galacturonic acid residues, hydroxyl groups	[37]
Algae	*Caulerpa racemosa*, *Sargassum muticum*	Functional groups of biomolecules in the cell extract adsorb and reduce AgNPs	Alginate, carboxyl groups, hydroxyl groups	[38,39]
Algae	*Hormophysa triquetra*	Functional groups of biomolecules in the cell extract adsorb and reduce AgNPs	Hydroxyl, amino, carboxyl groups in polyphenols, flavonoids, terpenoids, proteins, and lipids	[40]
Algae	*Porphyridium cruentum*	Functional groups of biomolecules in the cell extract adsorb and reduce AgNPs	Phycobiliproteins, hydroxyl, amino, carboxyl, and carbonyl groups	[41]
Algae	*Spirulina platensis*	Functional groups of biomolecules in the cell extract adsorb and reduce AgNPs	Phycobiliproteins, hydroxyl, amino, and carboxyl groups	[42]
Algae	*Nostoc linckia*	Functional groups of biomolecules in the cell extract adsorb and reduce AgNPs	Phycobiliproteins, hydroxyl and amino groups	[42]
Fungi	*Cladosporium halotolerans*, *Ganoderma sessile*	Functional groups of biomolecules in the cell-free extract adsorb and reduce AgNPs	Alcoholic and phenolic hydroxyl groups	[43,44]
Fungi	*Ganoderma lucidum*	Functional groups of biomolecules in the cell extract, adsorb, and reduce AgNPs	Carbonyl, hydroxyl groups	[45]
Fungi	*Trichoderma harzianum*, *Penicillium* sp.	Functional groups of biomolecules in the cell-free extract adsorb and reduce AgNPs	Amino groups	[44,46]
Fungi	*Fusarium oxysporum*, *Neurospora intermedia*, *Gymnascella dankaliensis*	Enzymatic reduction of AgNPs	Nitrate reductase, NADPH	[47,48,49]

**Table 2:** Summary of Bio-AuNPs synthesized by microorganisms.

Microorganism	Species	Main Mechanism	Key Molecules/ Functional Groups	References
Bacteria	*Marinobacter pelagius*	Functional groups in secretory proteins mediate the adsorption and reduction of AuNPs.	Primary and secondary amines, carbonyls, amino groups	[56]
Bacteria	*Lactiplantibacillus* sp., *Vibrio alginolyticus*	Functional groups in the cell-free extract mediate the synthesis and stabilization of AuNPs	Hydroxyl groups of phenolic and alcoholic compounds	[57,58]
Bacteria	*Streptomyces* sp.	Functional groups in proteins mediate the adsorption and reduction of AuNPs	Amino groups, hydroxyl groups	[59]
Bacteria	*Pseudomonas aeruginosa*	Functional groups on amino acid residues in peptidoglycans mediate the adsorption and synthesis of AuNPs	Carboxyl groups	[60]
Bacteria	*Delftia acidovorans*	Functional groups in secondary metabolites mediate the adsorption and synthesis of AuNPs	Delftibactin	[61]
Bacteria	*Escherichia coli*	Enzymatic reduction of AuNPs	Sulfite reductase, coenzyme α-NADPH	[62]
Bacteria	*Azospirillum brasilense*	Enzymatic reduction of AuNPs	Mn peroxidase, laccase	[63]
Bacteria	*Escherichia coli*	Enzymatic reduction of AuNPs	Reductases, Ausome	[64]
Algae	*Chondrus crispus*, *Gelidium corneum*, *Porphyra linearis*	Functional groups of biomolecules in the cell extract adsorb and reduce AuNPs	Carbonyl groups and sulfate moiety in protein, functional groups in polysaccharides	[65]
Algae	*Cystoseira baccata*, *Coelastrella thermophila*	Functional groups of biomolecules in the cell extract adsorb and reduce AuNPs	Hydroxyl groups in phenolic compounds, polysaccharides, and proteins	[66,67]
Algae	*Chlorella sorokiniana*	Functional groups of biomolecules in the cell extract adsorb and reduce AuNPs	amino groups	[68]
Algae	*Dunaliella salina*, *Spirulina* sp.	Functional groups of biomolecules in the cell extract adsorb and reduce AuNPs	Hydroxyl, carbonyl, and amine groups in enzymes, proteins, phenolic compounds, vitamins, and flavonoids	[69,70]
Algae	*Arthrospira platensis*	Functional groups in EPS mediate the adsorption and synthesis of AuNPs	Hydroxyl, amino and carboxyl groups	[71]
Algae	*Chlorella pyrenoidosa*, *Synechocystis* sp.	Electrons produced by photosynthesis reduce Au(III) to AuNPs	Electrons from water photolysis	[72,73]
Fungi	*Myriococcum thermophilum*	Enzymatic reduction of AuNPs	Cellobiose, dehydrogenase domain	[74]
Fungi	*Glomerella cingulata*	Enzymatic reduction of AuNPs	Glucose dehydrogenase	[75]
Fungi	*Candida rugopelliculosa*	Enzymatic reduction of AuNPs	NADH-dependent reductases	[76]
Fungi	*Flammulina velutipes*, *Lentinula edodes*	Functional groups of biomolecules in the cell extract adsorb and reduce AuNPs	Hydroxyl groups in polysaccharides and amide groups in proteins	[77,78]
Fungi	*Agaricus bisporus*	Functional groups of biomolecules in the cell extract adsorb and reduce AuNPs	Hydroxyl, amino, and ether groups on amino acids and polysaccharides	[79]
Fungi	*Alternaria chlamydospora*	Functional groups of biomolecules in the cell extract adsorb and reduce AuNPs	Hydroxyl, carboxyl groups	[80]

**Table 3:** Summary of Bio-PdNPs synthesized by microorganisms.

Microorganism	Strain Name	Main Mechanism	Key Molecules/ Functional Groups	References
Bacteria	*Geobacter sulfurreducens*	Extracellular electron transfer-mediated biosynthesis of PdNPs	Acetate, formate, hydrogen gas, outer-membrane c-type cytochromes	[94]
Bacteria	*Shewanella oneidensis*	Extracellular electron transfer-mediated biosynthesis of PdNPs	Electrons derived from anaerobic metabolism	[95,96]
Bacteria	*Staphylococcus saprophyticus*	Extracellular electron transfer-mediated biosynthesis of PdNPs	Hydroxyl groups (adsorption), riboflavin (electron shuttle), formate	[97]
Bacteria	*Citrobacter braakii*	Extracellular electron transfer-mediated biosynthesis of PdNPs	Electrons from anaerobic respiration	[98]
Bacteria	*Bacillus licheniformis*	Biosorption and polysaccharide-mediated bioreduction of Pd(II) to PdNPs	Oxygen- and nitrogen-containing functional groups, polysaccharides	[99]
Bacteria	*Providencia vermicola*	Functional groups in the cell wall adsorb Pd(II)	Carboxyl, hydroxyl, phosphate, amine groups	[100]
Bacteria	*Escherichia coli* (EC20)	Functional groups in the cell wall adsorb Pd(II)	Thiol group (-SH)	[101]
Algae	*Sargassum cervicorne*	Functional groups of biomolecules in the cell extract adsorb and reduce PdNPs	Alginate, carboxyl groups, hydroxyl groups	[102]
Algae	*Botryococcus braunii*, *Sargassum* sp.	Functional groups of biomolecules in the cell extract adsorb and reduce PdNPs	Polyphenols and polyols, hydroxyl groups	[103,104,105]
Algae	*Spirulina platensis*	Functional groups of biomolecules in the cell extract adsorb and reduce PdNPs	Hydroxyl, carboxyl, ester and ether groups	[106]
Algae	*Chlorella vulgaris*, *Asterarcys* sp.	Functional groups of biomolecules in the cell extract adsorb and reduce PdNPs	Amide and hydroxyl groups in polyols	[107,108,109]
Fungi	*Saccharomyces cerevisiae*	Functional groups in proteins and polysaccharides absorb and reduce Pd(II), and glutaraldehyde accelerates this process	Amines and carboxyl groups in proteins, glutaraldehyde	[110]
Fungi	*Neurospora crassa*	Functional groups in the cell wall adsorb and reduce Pd(II)	Phosphate, amino, hydroxyl, carboxyl groups	[24]
Fungi	*Phanerochaete chrysosporium*	Reductive protein-mediated bioreduction of Pd(II) to PdNPs	N and O atoms in amide groups, reductive proteins	[111]
Fungi	*Rhizopus* sp., *Saccharomyces cerevisiae*, *Agaricus bisporus*	Functional groups in secondary metabolites mediate the adsorption and synthesis of PdNPs	Hydroxyl groups	[112,113,114]

**Table 4:** Summary of Bio-PtNPs synthesized by microorganisms.

Microorganism	Strain Name	Main Mechanism	Key Molecules/ Functional Groups	References
Bacteria	*Acinetobacter calcoaceticus*	Enzymatic reduction of PtNPs	Intracellular reductases	[121]
Bacteria	*Bacillus* sp.	Enzymatic reduction of PtNPs	Nitrate reductases, NADH-dependent reductases	[122]
Bacteria	*Desulfovibrio vulgaris*	Enzymatic reduction of PtNPs	Periplasmic hydrogenases, H_2_	[123]
Bacteria	sulfate-reducing bacteria	Two hydrogenases mediate stepwise reduction: cytoplasmic to Pt(II), periplasmic to Pt(0)	Cytoplasmic hydrogenase, periplasmic hydrogenase	[124]
Bacteria	*Acidocella aromatica*, *Acidiphilium crytpum*	Enzymatic reduction of PtNPs	FDHs, formate, H_2_	[125]
Bacteria	*Escherichia coli* (BL21, EC20)	Functional groups in the cell wall adsorb Pd(II) and interact with proteins	Amino, hydroxyl, carboxyl, sulfhydryl, and phosphate groups, surface proteins	[126,127]
Bacteria	*Bacillus megaterium*	Reducing sugars from polysaccharide hydrolysis reduce Pt(IV)	Reducing sugars	[128]
Algae	*Sargassum polyphyllum*	Functional groups of biomolecules in the cell extract adsorb and reduce PtNPs	Hydroxyl and amino groups in biomolecules, ethyl 6,9,12- hexadecatrienoate	[129]
Algae	*Halymenia dilatata*	Functional groups of biomolecules in the cell extract adsorb and reduce PtNPs	Phenolic hydroxyl groups in polyphenols and flavonoids	[130]
Algae	*Calothrix pulvinate*, *Anabaena flos-aquae*	Enzymatic reduction of PtNPs	Nitrogenase, heterocysts and vegetative cells	[131]
Fungi	*Penicillium canescens*	Functional groups in secondary metabolites mediate the adsorption and synthesis of PtNPs	Secondary metabolites such as oxalic and citric acids	[132]
Fungi	*Streptomyces* sp.	Functional groups in secondary metabolites mediate the adsorption and synthesis of PtNPs	Amino groups	[133]
Fungi	*Alternaria alternata*	Enzymes and quinones reduce Pt(IV) to Pt(0)	Enzymes and quinones	[134]
Fungi	*Fusarium oxysporum*	Functional groups in secondary metabolites mediate the adsorption and synthesis of PtNPs	Hydroxyl-containing secretions, amino groups	[135,136]
Fungi	*Fusarium oxysporum*	Enzymatic reduction of PtNPs	Hydrogenase	[19]

**Table 5:** Summary of the applications of Bio-PMNPs.

Field	PMNPs	Application
Biomedical applications	Bio-AgNPs	Exhibit broad-spectrum antibacterial activity; Induce tumor cell apoptosis or activate immune responses; free-radical scavenging activity
	Bio-AuNPs	Induce apoptosis, generate oxidative stress, act as photothermal agents
	Bio-PdNPs	Photothermal bactericidal effects; broad-spectrum antimicrobial activity
	Bio-PtNPs	Mitochondrial dysfunction; induce apoptosis; selective cytotoxicity toward human breast cancer cells; Enhance radiation-induced DNA damage and tumor oxygenation; broad-spectrum antimicrobial activity; DPPH radical scavenging activities
Environmental remediation	Bio-AgNPs	Efficient degradation of organic pollutants, including 4-NP, malachite green, methyl orange, rhodamine B, and methylene blue
	Bio-AuNPs	Remove heavy metal ions Cr(VI); remove pesticides via chemisorption; degradation of organic pollutants, including 4-NP, phenol red, congo red, bromothymol blue, methylene blue
	Bio-PdNPs	Remove heavy metal ions Cr(VI); degradation of organic pollutants, including methyl orange, azo dyes, and recalcitrant halogenated organic pollutants: diatrizoate and trichloroethylene
	Bio-PtNPs	Remove heavy metal ions Cr(VI); remove refractory pharmaceutical contaminants such as sulfamethoxazole, ciprofloxacin, and endocrine-disrupting compounds
Catalysis	Bio-PdNPs	Efficiently catalyze Mizoroki–Heck, Suzuki–Miyaura, and Buchwald–Hartwig reactions, as well as the synthesis of N-aryl piperazine compounds and 2-substituted quinoline derivatives
Energy conversion and chemical sensors	Bio-AuNPs	Markedly enhance photoelectric conversion efficiency; function as highly efficient ‘electron conduits’ electrically coupling intracellular components with external electrodes, thereby facilitating extracellular electron transfer (EET); enhancing hydrogen production from algal cells under sunlight
	Bio-PdNPs	Used as anodic electrocatalysts in microbial fuel cells, enhancing power output; used to modify electrode surfaces in enzyme-free electrochemical sensors, enabling rapid and highly selective detection of H_2_O_2_

## Data Availability

No new data were created or analyzed in this study. Data sharing is not applicable to this article.
